# The Metabolites of *Lactobacillus fermentum* F-B9-1 Relieved Dextran Sulfate Sodium-Induced Experimental Ulcerative Colitis in Mice

**DOI:** 10.3389/fmicb.2022.865925

**Published:** 2022-04-28

**Authors:** Le Su, Feifan Ma, Zaiyong An, Xiuyu Ji, Ping Zhang, Qiulin Yue, Chen Zhao, Xin Sun, Kunlun Li, Baojun Li, Xinli Liu, Lin Zhao

**Affiliations:** ^1^State Key Laboratory of Biobased Material and Green Papermaking, School of Bioengineering, Qilu University of Technology, Shandong Academy of Sciences, Jinan, China; ^2^Shengshengxiangrong (Shandong) Biotechnology Co., Ltd., Jinan, China; ^3^Jinan Hangchen Biotechnology Co., Ltd., Jinan, China; ^4^Shandong Chenzhang Biotechnology Co., Ltd., Jinan, China

**Keywords:** ulcerative colitis, Caco-2, *Lactobacillus fermentum*, metabolites, gut microbiota

## Abstract

Because of the increased incidence and prevalence, ulcerative colitis (UC) has become a global health issue in the world. Current therapies for UC are not totally effective which result in persistent and recurrent symptom of many patients. *Lactobacillus* with anti-inflammatory effects might be beneficial to the prevention or treatment for UC. Here, we examined the ameliorative effects of the metabolites of *Lactobacillus fermentum* F-B9-1 (MLF) in Caco-2 cells and dextran sodium sulfate (DSS)-induced UC model mice. MLF displayed intestinal barrier-protective activities in Caco-2 cells by increasing the expression of Occludin and ZO-1. They also showed anti-inflammatory potential in interleukin (IL)-1β and IL-6. In order to further examine the *in vivo* anti-inflammatory effect of MLF, the MLF was gavaged in the DSS-induced UC model mice. The intragastric administration of MLF effectively alleviated colitis symptoms of weight loss, diarrhea, colon shortening, and histopathological scores, protected intestinal barrier function by increasing Occludin and ZO-1, and attenuated colonic and systemic inflammation by suppressing production of IL-1β and IL-6. Finally, the use of MLF remodeled the diversity of the gut microbiota and increased the number of beneficial microorganisms. Overall, the results demonstrated that MLF relieved DSS-induced UC in mice. And MLF might be an effective therapy method to UC in the clinic in the future.

## Introduction

Ulcerative colitis (UC) is an inflammatory disorder of the digestive tract that exists in the mucosa and submucosal areas (Sobczak et al., 2014). It initially affects the rectum, and then spreads to the innermost lining of the intestinal wall (Horsthuis et al., 2008; Wallace et al., 2014). Chronic, progressive, non-specific, uncontrolled, and recurrent are the characterized of UC (Zhou et al., 2019a,b). Although the etiology of UC is not fully elucidated, growing research shows that genetic predisposition, environmental factors, immune stimulation, and gut microbes play an important role (Iboshi et al., 2014; Jang et al., 2019; Zhou et al., 2019b). In recent decades, the incidence and prevalence of UC have been steadily increasing over the world. Many developing countries that have traditionally been considered low morbidity rates are undergoing a sharp rise in disease incidence and prevalence (Molodecky et al., 2012; Ng et al., 2013). UC is currently recognized as a global health issue.

The present treatment of UC is based on the control of the inflammatory responses, such as aminosalicylates antibiotics, corticosteroids, thiopurines, sulfasalazine, and biologicals (Sobczak et al., 2014). These therapies, however, are not effective in all patients. A number of patients require repeated surgeries to control disease complications with a narrow therapeutic window (Kwon and Farrell, 2005). Moreover, some of them are refractory to pharmacological interventions, which also may induce serious side effects (Ko and Auyeung, 2014). Even worse, biological therapies increase the risk of serious infection (Morrison et al., 2009). In recent years, there has been a lot of focus on finding new ways against UC.

It is well known that intake of beneficial bacterial species moderately could not only protect the host mucosa from unwarranted and potentially harmful inflammatory responses (Goyal and Shukla, 2013), but also stimulate the growth of beneficial bacterial species and restore dysregulated gut immune response (Fedorak and Madsen, 2004). Probiotics could work through a variety of mechanisms, including promotion of crypt cell proliferation (Ichikawa et al., 1999; Yan and Polk, 2002), increasing the expression of anti-inflammatory cytokines, and regulation of the intestinal immune system (Campieri and Gionchetti, 1999; Dieleman et al., 2003). *Lactobacillus fermentum* differs from other probiotic bacteria in that it produces antioxidants (glutathione) and short-chain fatty acids, as well as inducing the growth of other lactobacilli species (Peran et al., 2006, 2007). These characteristic properties play a positive role in regulation of intestinal homeostasis as well as IBD (Sun et al., 2017). In 2019, a new *L. fermentum* CQPC04 was isolated from traditional fermented pickles in Chongqing, China, which could effectively alleviate the symptoms of dextran sodium sulfate (DSS)-induced colitis mice (Zhou et al., 2019b). In the same year, Korean scientists also discovered that *L. fermentum* isolated from feces could weaken inflammatory diseases in the intestine by regulating the immune response and altering the composition of the intestinal microbiota (Jang et al., 2019). *Lactobacillus fermentum* might be used as an effective drug for UC in the future.

In this manuscript, *L. fermentum* F-B9-1 which was isolated from the soil was screened to investigate the *in vitro* anti-inflammatory activity in Caco-2 cells. We also confirmed our findings in a DSS-induced colitis animal model. The treatment was able to upregulate the expression of tight junction proteins and control the intestinal flora with no signs of toxicity, as well as ameliorate the levels of inflammatory factors *in vitro* and vivo systems that mimic UC.

## Materials and Methods

### Preparation of *Lactobacillus fermentum* F-B9-1

*Lactobacillus fermentum* F-B9-1 was isolated from the soil. The *L. fermentum* F-B9-1 was anaerobically grown at 37°C in De Mann, Rogosa, and Sharp (MRS) without shaking. The cultured bacterial strains were grown to an optical density between 4 and 5 at 600 nm (early stationary phase). The supernatants were collected *via* centrifugation at 12,000 × *g* for 30 min at 4°C, which were the metabolites of *L. fermentum* F-B9-1 named MLF (Shin et al., 2020). Immediately, the MLF was placed in a freeze dryer and frozen as solid. When we did the experiment, the sample was dissolved in milli-Q water.

### Separation of MLF

To isolate polysaccharides, trichloroacetic acid solution (20% v/v) was added into the MLF. The supernatant was added with two volumes of cold ethanol at 4°C for 12 h after centrifugation (12,000 × *g*, 30 min, 10°C). The exopolysaccharides were collected by centrifugation and dissolved in milli-Q water (Santiago-Lopez et al., 2018).

To isolate protein, ammonium sulfate was added to the MLF. The precipitate was collected by centrifugation at 12,000 × *g* to obtain crude protein. The precipitate was dialyzed in sterile water (3,000 KDa) to remove salt. The crude protein was purified by G-25 chromatographic column and frozen in a freeze dryer (Chon et al., 2010; Banik et al., 2018). Protein was dissolved in milli-Q water during using.

In order to get the lipids, methanol/chloroform (2:1) was added to metabolites. The chloroform is evaporated through the Eppendorf concentrator. The liquid was collected and frozen in a freeze dryer and dissolved in milli-Q water during using (Frick et al., 2007).

### Cell Culture and Treatment

Caco-2 cells were provided by the professor Yanqing Li from Qilu hospital of China (Jinan, China). The Caco-2 cells were cultured with Dulbecco’s Modified Eagle’s Medium-Hight glucose (DMEM-H; Gibco, 12800-017) supplemented with 10% heat-inactivated fetal bovine serum (FBS; v/v; HyClone, SV30087.02) and 1% penicillin/streptomycin (Genview, Lot: 010150101100). All cultures were incubated at 37°C under a humidified atmosphere of 5% CO_2_ and 95% air.

To determine the anti-inflammatory activities of MLF *in vitro*, Caco-2 cells were seeded on 24-well plates at a density of 1 × 10^5^ cells per well. When reached 70% confluency, cells were treated with lipopolysaccharide (50 μg/ml; LPS; Sigma-Aldrich, L4391) for 12 h. After that, Caco-2 cells were treated with different concentrations (0.25, 0.5, 1, and 2 mg/ml) of MLF or its fractions (exopolysaccharides, protein, or lipids) for 12 or 24 h.

### Cell Viability Analysis

Cell viability was assessed using 3-(4,5-dimethylthiazolyl-2)-2,5-diphenyltetrazolium bromide (MTT) assay. Cells were seeded in the 96-well plate at a density of 1 × 10^4^ cells per well. Cells were incubated with or without stimuli with MLF for 24 or 48 h. After 4 h of incubation with MTT, 150 μl dimethylsulfoxide (DMSO) was added to each well. The plate was read using absorbance at 570 nm by the SpectraMax ABS microplate spectrophotometer (Molecular Devices, United States). The percentage of living cells was calculated by the ratio of OD.

### Animal Treatment

In this experiment, we utilized 6–8-week-old male C57BL/6 mice (20–24 g), which were provided by Beijing Vital River Laboratory Animal Technology Co., Ltd. Mice were housed under specific pathogen-free conditions at 22–23°C for 6 days before the experiment. And mice were maintained on 12/12 h light/dark cycles with water and food *ad libitum*. All animal experiments complied with the ARRIVE guidelines and were carried out in accordance with the United Kingdom Animals (Scientific Procedures) Act, 1986 and associated guidelines, EU Directive 2010/63/EU for animal experiments, and the National Institutes of Health guide for the care and use of Laboratory animals (NIH Publications No. 8023, revised 1978). The animal experimental protocol complied with the Animal Management Rules of the Chinese Ministry of Health (document no. 55, 2001) and was approved by the Animal Experiment Ethics Committee of Qilu University of Technology. Mice were randomly assigned to six groups, each containing seven mice, as follows: normal control group (NOR), DSS (molecular weight: 40000; Shanghai Aladdin Biochemical Technology Co., LTD, China) group (DSS) and different doses of MLF supplemented groups (0.05, 0.1, 0.2, and 0.4 g/kg/day; MLF). For colitis development, the mice in DSS group and MLF groups were exposed to 3% DSS uninterruptedly in drinking water on days 7–11. The mice in MLF groups were treated with MLF by the method of gavage on days 12–19. At other times, the other mice drank water normally. At day 20, the mice were euthanized by cervical dislocation (Yokota et al., 2018). The mice were weighed and recorded at the same time daily.

### Disease Activity Index Assessment

The disease activity index (DAI) scores were calculated blindly according to the Hamamoto et al. (1999). Body weight, fecal characteristics, and occult blood test results were recorded every day.

### Blood and Tissue Collection

Serum was prepared by centrifugation at 3,000 × *g* for 20 min at 4°C and stored at −80°C for biochemical analysis. The colons were removed aseptically after mice were killed by exsanguination. We measured and recorded the length of the colon of each mouse. All samples were stored at −80°C.

### Histopathological Analysis

For assessment of macroscopic damage, colonic segments (approximately 2–3 cm long) were excised, flushed with PBS, and placed in 10% buffered formalin for 24 h. The colonic segments were immersed in OCT embedding medium followed by microtomy. Colon sections (5 μm) were obtained and stained with hematoxylin and eosin (H&E staining). Each slice was subjected to double-blind histological damage scoring using the Dieleman integral standard (Dieleman et al., 1998).

### Enzyme-Linked Immunosorbent Assays

Cell supernatants and serum were collected respectively for the determination of IL-6, IL-1β, and TNF-α by ELISA kit according to the manufacturer’s instructions (Beijing dakowei Biotechnology Co., Ltd., Beijing, China).

### Real-Time Reverse Transcription–PCR

Total RNA was extracted from the Caco-2 cells and colon tissue using TRIzol (Thermo Fisher Scientific Co., Ltd., United States) at low temperatures. And cDNA was generated using a commercial real-time reverse transcription PCR (RT-PCR) kit (ABclonal Co., Ltd., Wuhan, China). Then, real-time PCR was conducted using the SYBR Green Quanti Tect RT-PCR kit (ABclonal Co., Ltd., Wuhan, China) and each of the samples was analyzed in triplicate. The sequences of the primers used are shown in Table 1. For each sample, Glyceraldehyde-3-phosphate dehydrogenase (GAPDH) was used as an internal reference. The relative mRNA expression of the target genes was calculated by the 2^−∆∆Ct^ method.

### Western Blotting

Caco-2 cells were treated with LPS for 12 h and treated with MLF (1 and 2 mg/ml) for 24 and 48 h. Then, the cells were lysed with a lysis buffer (Beyotime, Beijing, China). The protein concentrations in the supernatants were quantified using a bicinchoninic acid protein assay kit (Beyotime, Beijing, China). Around 15 μg protein was resolved by 4–15% gradient gel (Bio-Rad Laboratories, United States). The membrane was probed with primary antibodies against ZO-1 (1:5,000; ABclonal Co., Ltd., Lot: 3560390003), Occludin (1:5,000) (ABclonal Co., Ltd., Lot: 0026860201) and β-actin (1:2,000; Sigma-Aldrich, USA; A5441). Then, the membranes were incubated with a horseradish peroxidase-labeled secondary goat anti-rabbit (1:2,000) or rabbit anti-mouse (1:2,000; ABclonal Co., Ltd., Wuhan, China) antibody for 1 h at room temperature. Next, the membranes were visualized using enhanced chemiluminescence reagent (ECL kit; Millipore Co., Ltd., United States) by the Amersham Imager 600 (General Electric Co., Ltd., United States).

### 16S RNA Analysis

The feces from cecum were collected and stored at −80°C. A study on the composition spectrum of intestinal microbial diversity was performed by Personal Biotechnology Technology Co., Ltd (Shanghai, China). The sequences were clustered into operational taxonomic units of at least 97% identity, and the relative abundances of the microbial taxa (genus to phylum) were generated from non-rarefied operational taxonomic unit tables. Species richness (alpha diversity) was measured using the Shannon index. Beta diversity was calculated using the UniFrac distance between samples and visualized in three-dimensional plots based on a weighted principal coordinate analysis.

### Data Analysis and Statistics

All experiments were repeated at least three times independently. The normal distribution was firstly analyzed by SPSS v11.5 (SPSS Inc., Chicago, IL). All the data were expressed as mean ± SEM and analyzed with one-way ANOVA with use of SPSS v11.5 to compare the treatment means when data were normally distributed. Tukey–Kramer multiple comparison procedure was used for *post-hoc* comparisons. Values of *p* < 0.05 were considered statistically significant. Images were processed by use of GraphPad Prism 5 (GraphPad Software, La Jolla, CA, United States) and Adobe Photoshop CS6 (Adobe, San Jose, United States).

## Results

### Anti-inflammatory Effects of MLF in Caco-2 Cells

**Figure 1:**
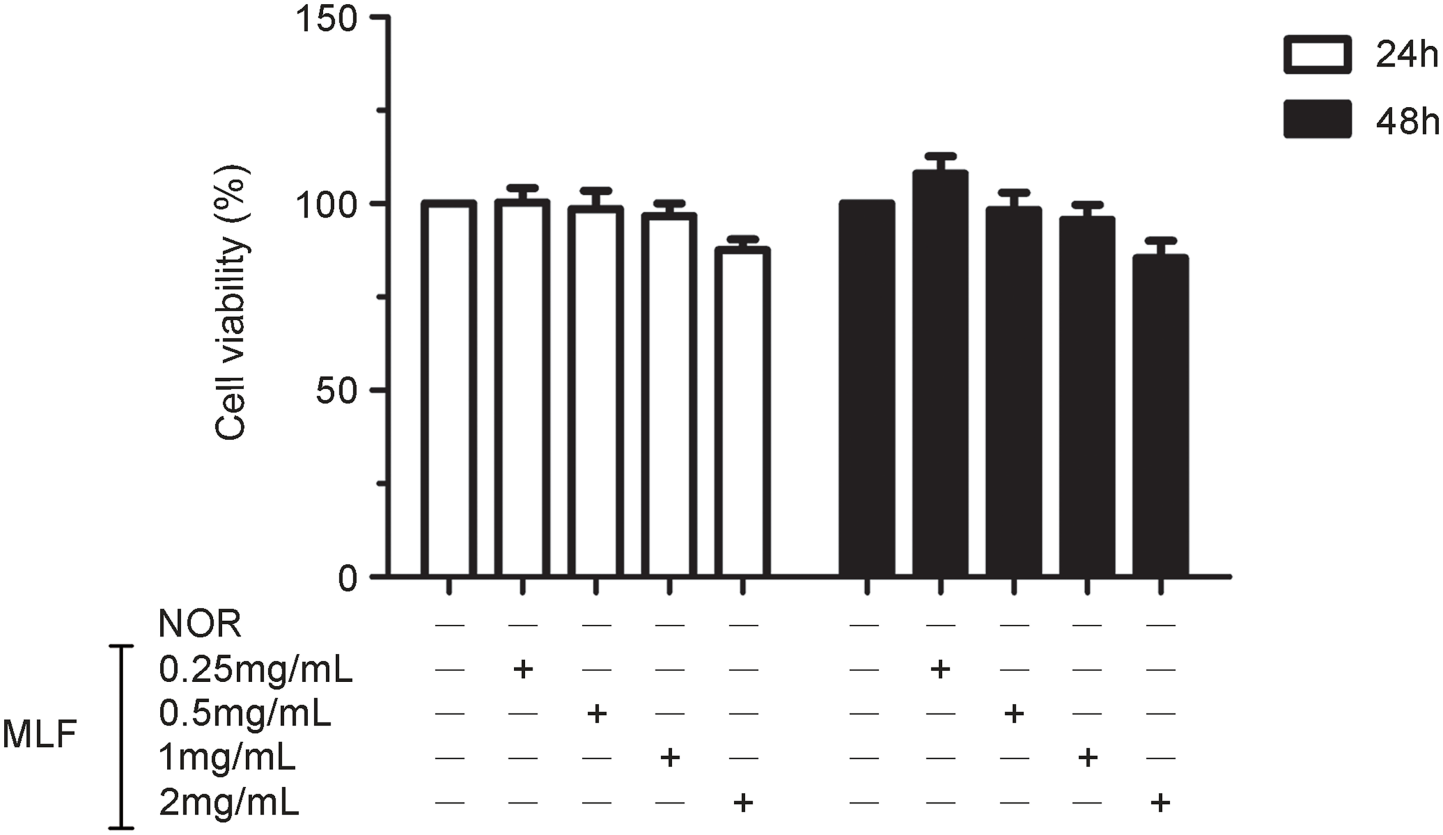
Effects of metabolites of *Lactobacillus fermentum* F-B9-1 (MLF) on the cell viability of Caco-2 cells detected by 3-(4,5-dimethylthiazolyl-2)-2,5-diphenyltetrazolium bromide (MTT). Caco-2 cells were treated with different concentrations (0.25, 0.5, 1, and 2 mg/ml) of MLF for 24 and 48 h. The OD were examined in Caco-2 cells which were pretreated with 5 mg/ml MTT for 4 h, then treated with dimethylsulfoxide (DMSO).

Firstly, we investigated the effect of MLF on cell viability. The MTT test revealed that there was no significant change in cell viability after treating Caco-2 cells with MLF (0.25, 0.5, 1, and 2 mg/ml) for 24 or 48 h (Figure 1). MLF had no significant cytotoxicity in Caco-2 cells. As a result, these four doses of MLF were safely used in the cellular experiments below.

**Table 1:** Primer sequences used for real-time RT-PCR.

Target genes	Forward(F)/Reverse(R)	Sequence	Size (bp)
H-GAPDH	F R	CAACGACCACTTTGTCAAGC TTCCTCTTGTGCTCTTGCTG	140
H-IL-1β	F R	GCAGAAGTACCTGAGCTCGCC CCTTGCTGTAGTGGTGGTCGG	175
H-IL-6	F R	CACTGGTCTTTTGGAGTTTGAG GGACTTTTGTACTCATCTGCAC	101
H-TNF-α	F R	AGCTGGTGGTGCCATCAGAGG TGGTAGGAGACGGCGATGCG	124
M-GAPDH	F R	TGTGTCCGTCGTGGATCCGA TTGCTGTTGAAGTCGCAGGAG	150
M-IL-1β	F R	GCCACCTTTTGACAGTGATGAG ATGTGCTGCTGCGAGATTTG	135
M-IL-6	F R	ACCCCAATTTCCAATGCTCTCC GCATAACGCACTAGGTTTGCC	148
M-TNF-α	F R	GGACTAGCCAGGAGGGAGAACAG GCCAGTGAGTGAAAGGGACAGAAC	103
M-Occludin	F R	TGGCTATGGAGGCGGCTATGG AAGGAAGCGATGAAGCAGAAGGC	114
M-Z0-1	F R	GCGAACAGAAGGAGCGAGAAGAG GCTTTGCGGGCTGACTGGAG	112

**Figure 2:**
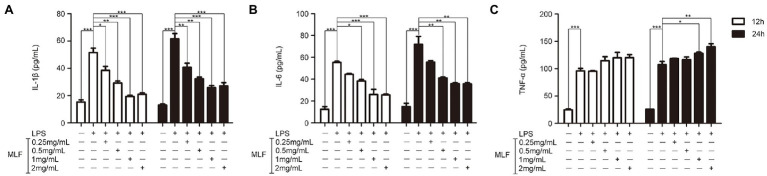
The protein levels of IL-1β, IL-6, and TNF-α in caco-2 cells. IL-1β **(A)**, IL-6 **(B)**, and TNF-α **(C)** levels were examined in the supernatant of Caco-2 cells which were pretreated with 50 μg/ml lipopolysaccharide (LPS) for 12 h, then treated with MLF (0.25, 0.5, 1, and 2 mg/ml) for 12 or 24 h. All groups were statistically analyzed with the LPS group. Data are expressed as means ± S.E. ^*^p < 0.05, ^**^p < 0.01, and ^***^p < 0.001.

**Figure 3:**
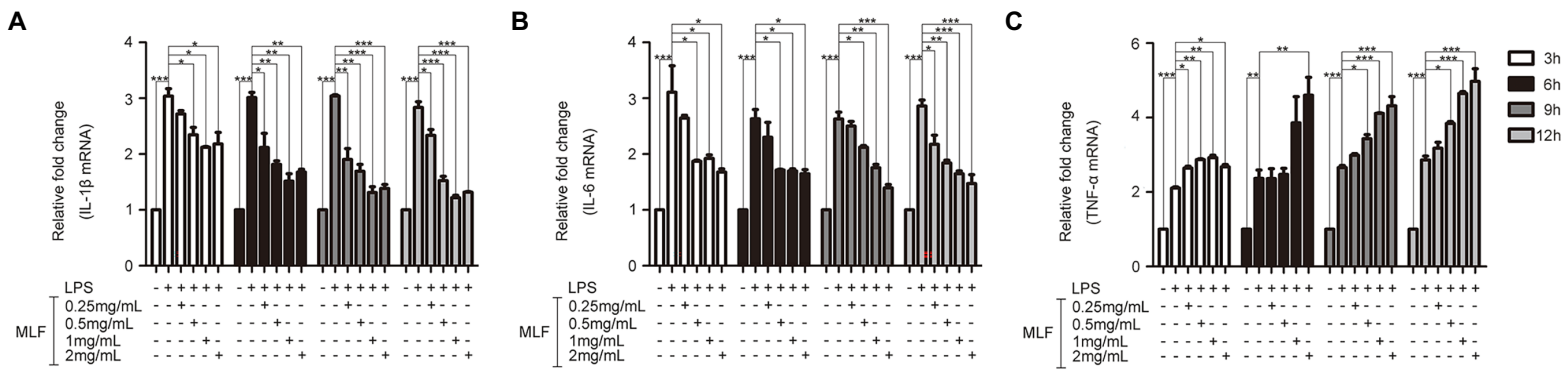
The mRNA levels of IL-1β, IL-6, and TNF-α in caco-2 cells. IL-1β **(A)**, IL-6 **(B)**, and TNF-α **(C)** mRNA levels were examined in Caco-2 cells which were pretreated with 50 μg/ml LPS for 12 h, then treated with MLF (0.25, 0.5, 1, and 2 mg/ml) for 3, 6, 9, or 12 h. All groups were statistically analyzed with the LPS group. Data are expressed as means ± S.E. ^*^p < 0.05, ^**^p < 0.01, and ^***^p < 0.001.

The effects of MLF on pro-inflammatory cytokines were investigated in cellular supernatant by ELISA. As shown in Figure 2, the levels of IL-1β, IL-6, and TNF-α in Caco-2 cells were all upregulated after being treated with LPS. The levels of IL-1β were decreased in all MLF groups compared with that in LPS group (Figure 2A). The levels of IL-6 were inhibited in 0.5, 1, and 2 mg/ml MLF groups compared with that in the LPS group (Figure 2B). However, MLF could not inhibit the level of TNF-α induced by LPS in Caco-2 cells (Figure 2C). The data from RT-PCR assay showed the similar results. As shown in Figure 3, the mRNA levels of IL-1β, IL-6, and TNF-α were all upregulated after being treated with LPS. MLF at 0.5, 1, and 2 mg/ml suppressed the mRNA levels of IL-1β and IL-6 significantly (Figures 3A,B). However, MLF could not suppress the mRNA level of TNF-α which was consistent with the ELISA results (Figure 3C). These data indicated that MLF had anti-inflammatory capacity *in vitro*.

### The Effects of MLF on Tight Junction Proteins in Caco-2 Cells

**Figure 4:**
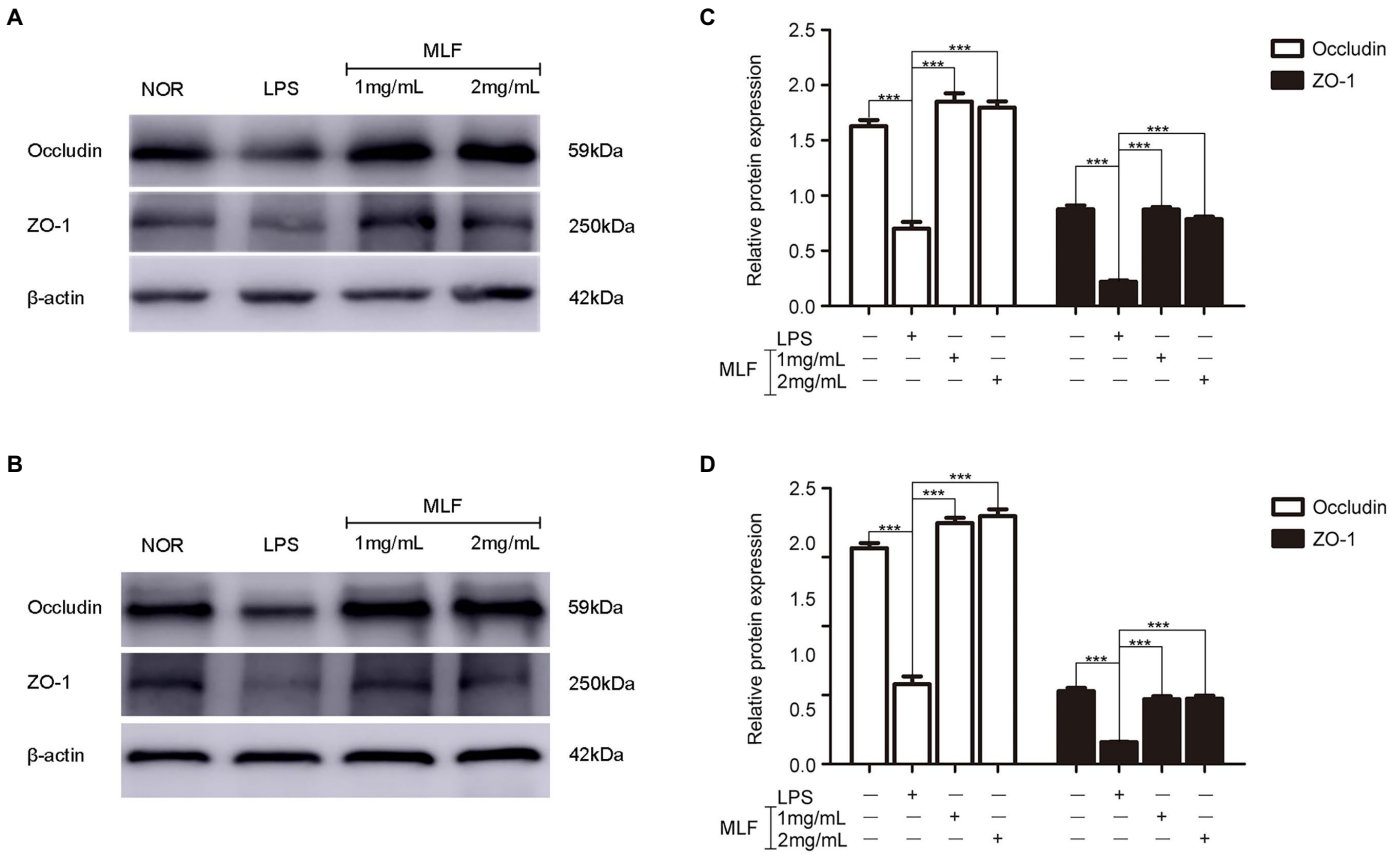
The levels of Occludin and ZO-1 in caco-2 cells. Occludin and ZO-1 levels were examined in Caco-2 cells which were pretreated with 50 μg/ml LPS for 12 h, then treated with MLF (1 and 2 mg/ml) for 24 h **(A)** or 48 h **(B)**. **(C,D)** quantification of Occludin and ZO-1. All groups were statistically analyzed with the LPS group. Data are expressed as means ± S.E. ^***^p < 0.001.

Western blotting results showed that the levels of Occludin and ZO-1 were decreased in the LPS group (Figures 4A,C). MLF (1 and 2 mg/ml) significantly elevated the expression of two typical tight junction proteins (Figures 4B,D). MLF played an important role in the enhancement of tight junction integrity in Caco-2 cells.

### MLF Alleviated the Clinical Symptoms of DSS-Induced Colitis in Mice

**Figure 5:**
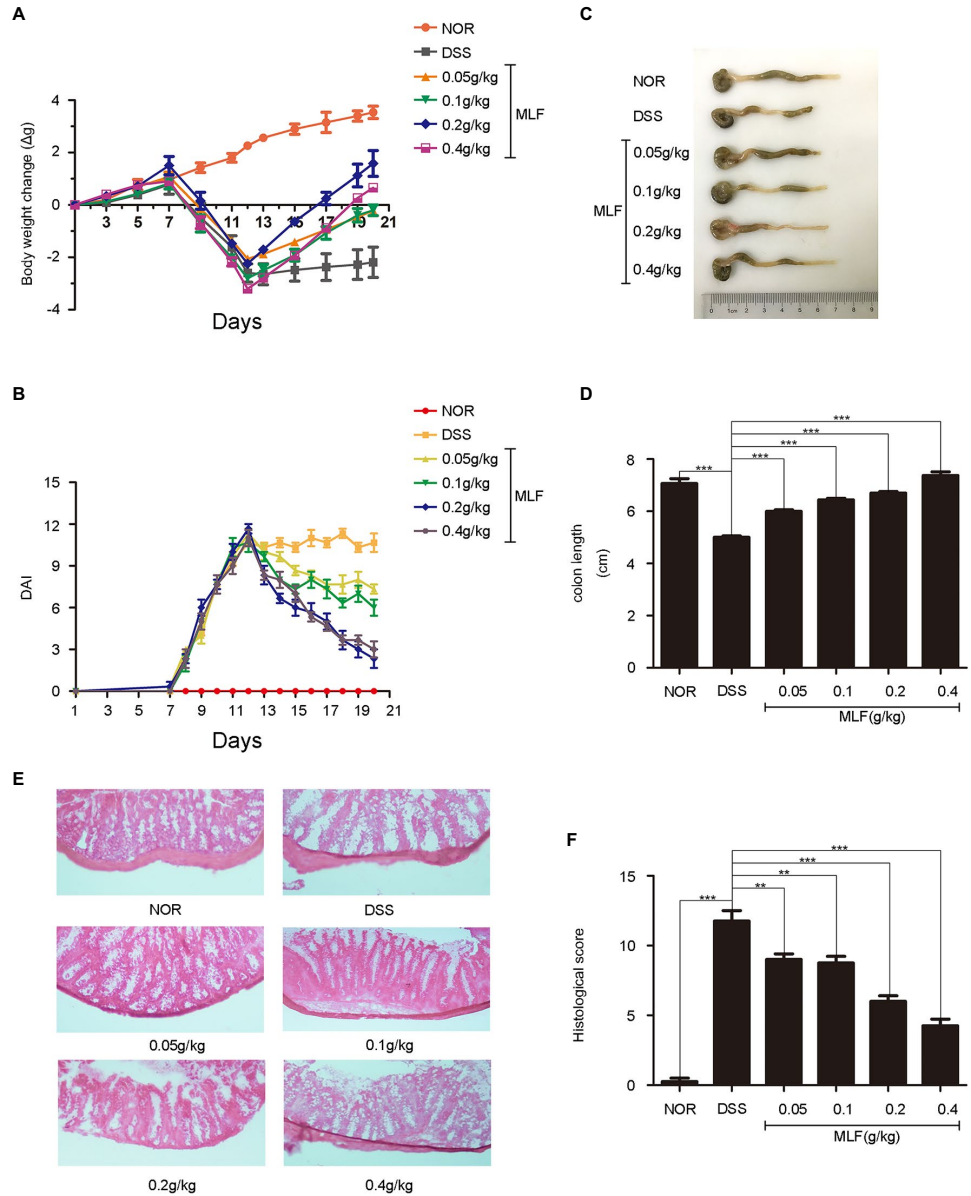
Improvement of symptoms in dextran sodium sulfate (DSS)-induced colitis by MLF. **(A)** The body weight change was evaluated throughout the experiment and expressed as the weight change relative to the previous day. **(B)** Disease activity index (DAI) score was examined every day. **(C)** The photos of colon length (one representative colon from each group). **(D)** The colon lengths calculated from **(C)**. All groups were statistically analyzed with the DSS group. **(E)** The distal colon was stained with hematoxylin and eosin to determine the degree of inflammation, and **(F)** the histological score was measured. Data are expressed as means ± S.E. ^**^p < 0.01 and ^***^p < 0.001.

Figure 5A depicted the changes in the mice’s body weights during the 20 days of experimental period. The DAI of the mice changed during the course of the 20 days, as seen in Figure 5B. All animals that were treated with DSS showed a significant decrease in body weight immediately afterward compared with control animals. The MLF-treated mice had notably less weight loss than the DSS group, and the effect of 0.2 g/kg dose and 0.4 g/kg dose MLF was more significant (Figure 5A). The DAI score was used to judge the severity of UC. The DAI score combined score of weight loss, stool consistency, and fecal blood. The DSS treatment caused a rise in the DAI, and this was attenuated by intragastric administration of MLF (Figure 5B). These results indicated that MLF effectively relieved DSS-induced UC symptoms.

The colon length was a biological marker for the severity of colitis in mice. Colon length was shortened in all DSS-treated mice. The colon length of the MLF treatment group showed a significant increase compared with the DSS group (Figures 5C,D). When the concentration of MLF was 0.4 g/kg, the colon length was almost returned to the normal level. The data suggested that the MLF alleviated the effects of DSS on colon length shortening.

As shown in Figure 5E, the result showed typical images of HE-stained colonic tissue. The colonic tissue from DSS-treated mice showed severe epithelial damage, the loss of colonic epithelial cells, distortion of crypt structure, massive inflammatory cell infiltration, and inflammatory lesions throughout the mucosa and submucosa. As shown in Figure 5F, mice only with DSS had the highest histological score. By contrast, compared with the DSS-only treatment group, the colons in MLF-treated mice showed ameliorated structural damage and exhibited less inflammatory cell infiltration and only mild evidence of crypt distortion. This was in line with the result of the histological score. The ameliorative effects of MLF on DSS-induced mice were also observed in histological injury scores. Taken together, our results suggested that MLF significantly protected colon tissue and attenuated DSS-induced tissue morphological changes.

### The Effects of MLF on Pro-inflammatory Cytokines in Mice

**Figure 6:**
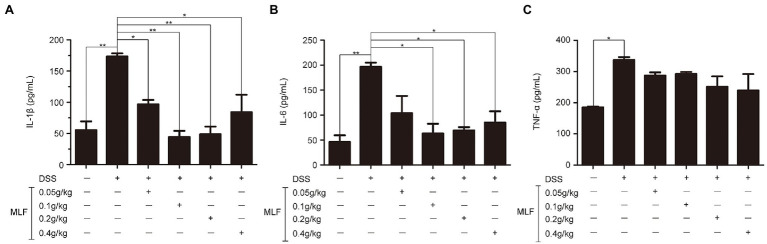
Effects of MLF on the levels of IL-1β, IL-6, and TNF-α. The levels of IL-1β **(A)**, IL-6 **(B)**, and TNF-α **(C)** in serum of DSS-induced UC mice were detected using ELISA Kits. MLF groups were statistically analyzed with the DSS group. Data are expressed as means ± S.E. ^*^p < 0.05 and ^**^p < 0.01.

**Figure 7:**
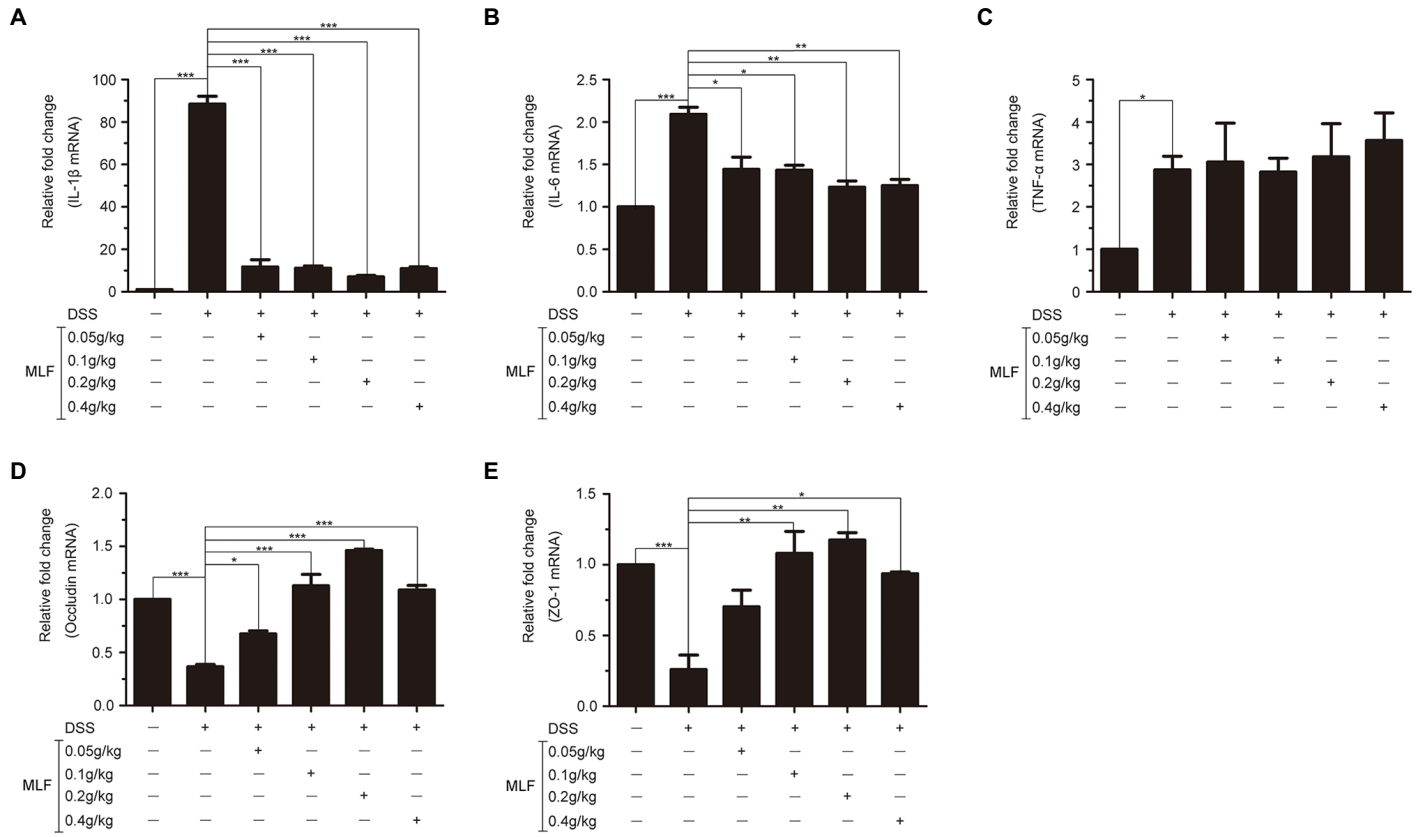
Effects of MLF on the mRNA levels of inflammatory cytokines and tight junction protein in mice. The levels of IL-1β **(A)**, IL-6 **(B)**, TNF-α **(C)**, Occludin **(D)**, and ZO-1 **(E)** in colon of DSS-induced UC mice were detected using real-time reverse transcription PCR (RT-PCR). MLF groups were statistically analyzed with the DSS group. Data are expressed as means ± S.E. ^*^p < 0.05, ^**^p < 0.01, and ^***^p < 0.001.

The effects of MLF on pro-inflammatory cytokines were investigated in mice serum and colon tissues by ELISA and RT-PCR. As shown in Figures 6, 7, the levels of IL-1β, IL-6, and TNF-α were induced after DSS treatment. The levels of IL-1β and IL-6 were attenuated in the 0.1, 0.2, and 0.4 g/kg MLF groups (Figures 6A,B). Compared with the DSS group, the 0.05, 0.1, 0.2, and 0.4 g/kg of MLF also suppressed the mRNA levels of IL-1β and IL-6 (Figures 7A,B). However, the levels of TNF-α were not inhibited both in serum and colon tissues which was consistent with the *in vitro* results in Caco-2 cells (Figures 6C, 7C).

### The Effects of MLF on Tight Junction Proteins in Mice Colon Tissues

The effects of MLF on tight junction proteins were investigated in mice colon by RT-PCR. As shown in Figures 7D,E, the levels of Occludin and ZO-1 in colon tissues were reduced in DSS group. MLF restored the reduction. The levels of Occludin and ZO-1 were improved in 0.1, 0.2, and 0.4 g/kg MLF groups. The above data indicated that MLF not only had the anti-inflammatory capacity, but also could improve the levels of tight junction. The MLF at 0.1, 0.2, and 0.4 g/kg were more effective than that at 0.05 g/kg MLF.

### MLF Improved DSS-Induced Dysbiosis of the Gut Microbiota

**Figure 8:**
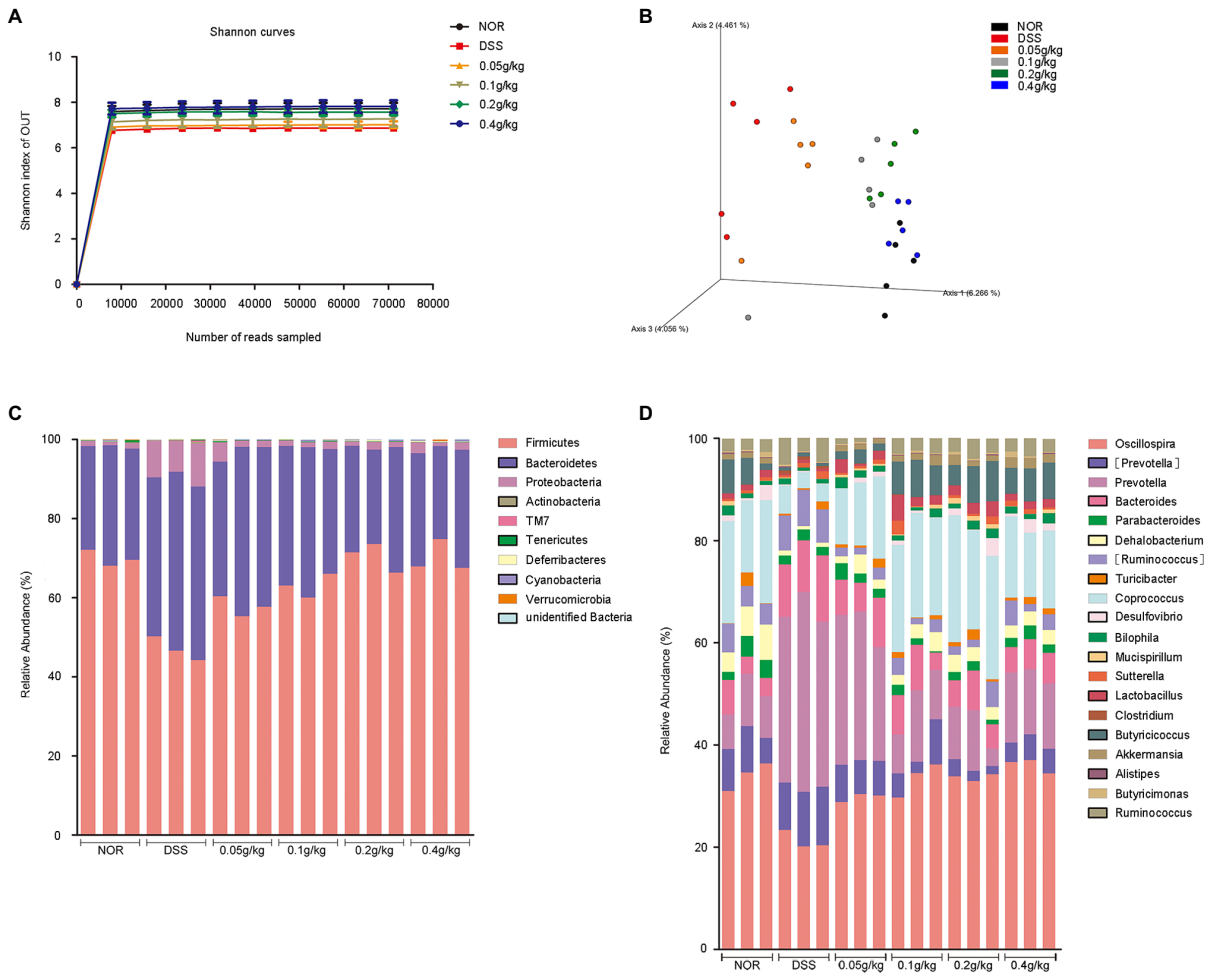
Taxonomic changes in the microbiota of the mouse cecum induced by MLF. A microbiome analysis was conducted using cecal samples collected from five mice per group. **(A)** Rarefaction curves for each group calculated at OTU level of sequence similarity clustering. **(B)** Principal coordinates analysis of the cecal microbiota structure measured by weighted UniFrac distance. **(C)** Average relative abundances of taxa at the phylum level. **(D)** Average relative abundances of taxa at the genus level.

We investigated the effects of MLF intake on the cecal microbiome abundance and diversity using a 16S RNA sequencing analysis to further explore the mechanisms of ameliorative effects of MLF. Alpha diversity was analyzed by calculating the Shannon indices (Figure 8A). The rarefaction curves for all mice reached a plateau, indicating that the bacterial diversity in these communities was mostly covered. The decrease in the number of OUT also indicated the dysbiosis of the intestinal microbes in mice administered DSS. MLF treatment completely reversed the altered diversity, it increased the microbial diversity in some extent. The bacterial communities of the same groups in mice were more likely to cluster together, but there were differences in the gut microbiota patterns among the six groups (Figure 8B). The difference between NOR and DSS groups was the most significant. The use of MLF had gradually narrowed the difference. The higher the concentration of MLF, especially the 0.4 g/kg, the closer the bacterial communities were to the NOR group.

The cartogram of the microbiota at the phylum level was shown in Figure 8C. The treatment of DSS increased the proportions of *Proteobacteria* and *Bacteroidetes* and decreased the proportion of *Firmicutes* compared with those in the NOR group. The presence of MLF reversed these changes. The relative proportion of *Bacteroidetes* was nearly the same among the groups after the use of MLF. At the genus level (Figure 8D), compared with the DSS group, MLF selectively blunted the expansion of the *Prevotella* and *Ruminococcus*. However, the diversity had changed in the MLF group compared with the DSS group mice, like *Clostridium* and *Akkermansia*. At the same time, the use of MLF increased the relative proportion of *Coprococcus*, *Oscillospira*, *Lactobacillus*, *Butyricicoccus*, and *Dehalobacterium*, whereas the other major families were only marginally affected.

Collectively, based on these data, MLF treatment played essential roles in alleviating DSS-induced colitis by maintaining microbiome abundance and diversity.

### Exopolysaccharides and Protein Might Be the Active Constituents for the Inflammation Inhibition

**Figure 9:**
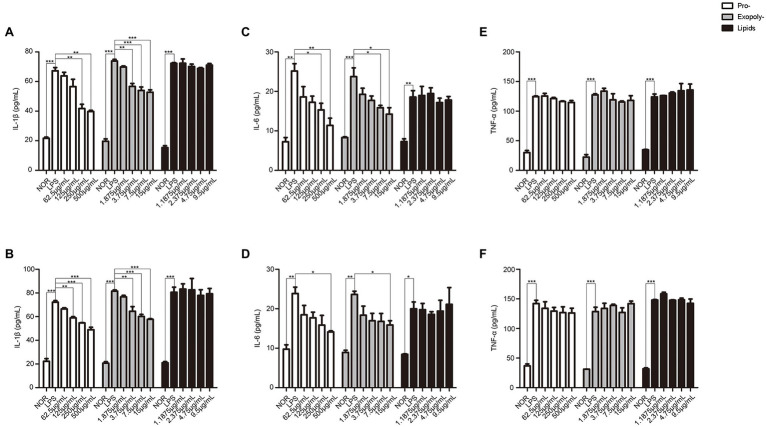
The levels of IL-1β, IL-6, and TNF-α in caco-2 cells after treated with exopolysaccharides, protein, and lipids separated from MLF. IL-1β **(A)**, IL-6 **(B)**, and TNF-α **(C)** levels were examined in the supernatant of Caco-2 cells which were pretreated with 50 μg/ml LPS for 12 h, then treated with exopolysaccharides, protein, or lipids for 12 h. IL-1β **(D)**, IL-6 **(E)**, and TNF-α **(F)** levels were examined in the supernatant of Caco-2 cells which were pretreated with 50 μg/ml LPS for 12 h, then treated with exopolysaccharides, protein, and lipids for 24 h. All groups were statistically analyzed with the LPS group. Data are expressed as means ± S.E. ^*^p < 0.05, ^**^p < 0.01, and ^***^p < 0.001.

Finally, in order to identify which components in MLF had the effect on UC, exopolysaccharides, protein, and lipids were separated from MLF. The data showed that the levels of IL-1β and IL-6 induced by LPS were only inhibited in the exopolysaccharides or protein treatment groups. All the exopolysaccharides, protein, and lipids could not decrease the levels of TNF-α (Figure 9). Thus, exopolysaccharides and protein might be the active constituents for the inflammation inhibition.

## Discussion

An accumulation of evidence indicated that *L. fermentum* could relieve inflammation in UC (Geier et al., 2007; Santiago-Lopez et al., 2018; Zhou et al., 2019b). For example, *L. fermentum* CQPC04 could inhibit the release of pro-inflammatory cytokines TNF-α, IFN-γ, IL-1β, IL-6, and IL-12 (Zhou et al., 2019b). *Lactobacillus fermentum* BR11 could effectively alleviate the colon shortening and the crypt hyperplasia in mice (Geier et al., 2007). However, these reports all focused on the anti-inflammatory effects of *L. fermentum*. The potential influence of intestinal epithelial barrier and gut microbiota on UC was ignored. The efficacy of MLF in pro-inflammatory cytokines, intestinal epithelial barrier, and intestinal flora was studied in this manuscript.

The levels of IL-1β, IL-6, and TNF-α dramatically increased in the serum and colonic tissue of the DSS treatment group. IL-1β could promote the macrophages and monocytes to secrete a variety of pro-inflammatory cytokines, it played an key role in the pathogenesis of colitis (Min et al., 2020). As a pleiotropic pro-inflammatory cytokine, IL-6 played a key role in host defense against pathogens and acute stress (Yao et al., 2014). Some researchers suggested that blocking the expression of IL-1β and IL-6 could become an important direction for UC (Yan et al., 2019). The cytokine concentration in serum represented the inflammation of the whole body (Yan et al., 2019). *In vivo*, the following indicators could indicate the severity of UC directly: body weight, DAI score, colon length, and histological score. In this study, MLF treatment reduced the levels of IL-1β and IL-6, which would release into the serum. The levels of pro-inflammatory cytokines in mice’s serum were lowered, as expected. Meanwhile, MLF alleviated the symptoms of colonic inflammation induced by DSS, which included body weight, bloody stool, and poor stool consistency, thereby reducing the DAI score. MLF also reduced the infiltration of neutrophils and inflammatory cells in the colon. As reported in previous studies, DSS-induced colitis was characterized by a significant increase in neutrophils and inflammatory cells in the colon (Bian et al., 2020). These reflected the powerful anti-inflammatory activity of MLF, including the effects of MLF on cell models. Inhibited the levels of inflammatory cytokine might be a possible mechanism by which MLF reduced the inflammatory level in colitis.

Ulcerative colitis was thought to be associated with increased intestinal permeability caused by the decrease of the expression of tight junction proteins in the mucosa with inflammation. In UC patients, epithelial barrier function was known to be disrupted, and those with a higher degree of intestinal permeability had been shown to be at a greater risk of relapse. An impaired intestinal barrier could exacerbate diarrhea and increase the infiltration of pro-inflammatory cytokines, thereby stimulating the underlying immune system, enhancing and maintaining chronic intestinal inflammation (Bailey et al., 2017). ZO-1 and Occludin were the two main proteins that form the core part of the tight junction and regulated ion selectivity and paracellular permeability (Schneeberger and Lynch, 2004). MLF restored the levels of ZO-1 and Occludin in our study as compared to the DSS group. This was also confirmed by the restoration of crypt structure in the mouse colon. MLF had the ability to repair the leakage of the intestinal epithelial barrier and maintain the integrity of intestinal epithelial barrier. This was consistent with DAI and histological scores. Therefore, the UC-ameliorating effect of MLF was partly attributed to its ability to preserve the integrity of the intestinal epithelial structure.

Many studies had shown that the disorder of inflammatory cytokines could lead to the imbalance of the gut microbiota. The integrity of the intestinal barrier would be harmed by an imbalance of the gut microbiota. Probiotics, such as *L. fermentum*, had been shown to affect the production of inflammatory cytokines (Zhang et al., 2019), adjust the abundance and diversity of gut microbiota dysbiosis, and correct the abnormal reaction of the mucosal immune system to intestinal inflammation (Rath et al., 2001; Kim et al., 2017; Ni et al., 2017), thereby enhancing the microbial barrier function. In the present study, we observed broad gut microbiota dysbiosis, including decreasing in OTU numbers and disordering in beta diversity. In UC patients, the dispersion of microorganisms was much fewer than healthy subjects (Lo Presti et al., 2019). MLF could help to restore the gut microbiota dysbiosis in DSS-induced colitis mice. The condition of the human gut microbiota had been shown to be reflected in the ratio of *Firmicutes* to *Bacteroidetes* (Almagro-Moreno and Boyd, 2009; Zhang et al., 2019). The use of MLF increased the ratio of *Firmicutes* to *Bacteroidetes*. *Proteobacteria* were also a possible indicator of disease onset in the mouse model of DSS-induced colitis, and its abundance decreased upon recovery of the mice (Almagro-Moreno and Boyd, 2009; Munyaka et al., 2016). The use of MLF promoted the growth of beneficial bacteria and minimized harmful bacteria in the gut microbiota. At the genus level, the most variable bacteria were *Prevotella* and *Oscillospira* in the gut microbiota. Th17-mediated mucosal inflammation was related to the increased in the abundance of *Prevotella* (Larsen, 2017). The results led to massive inflammatory cell infiltration and inflammatory lesions in colon tissue of DSS-induced mice. In addition, *Prevotella* could activate TLR2 leading to production of Th17-polarizing cytokines by antigen-presenting cells, including IL-1. And *Prevotella* could also stimulate epithelial cells to release large amounts of IL-6. Usually, *Oscillospira* and *Lactobacillus* were reduced in UC patients. *Oscillospira* and *Lactobacillus* could protect the host mucosa from unwarranted and potentially harmful inflammatory responses. The use of MLF reversed the reduction of *Oscillospira* and *Lactobacillus*. Compared with the DSS group, the *Akkermansia* reappeared in the MLF group. *Akkermansia* could degrade mucin into short-chain fatty acid, subsequently regulating host immune responses and biological functions (Bian et al., 2020). In addition, *Akkermansia* could also reduce IL-6 production. These strains would increase the integrity of the mucus layer and maintain the integrity of the intestinal barrier, thereby alleviating DSS-induced colitis (Everard et al., 2013). The use of MLF led to the disappearance of *Clostridium*. *Clostridium* was a harmful bacteria that could cause a spectrum of diseases ranging from mild or moderate diarrhea to fulminant infectious colitis (D'Aoust et al., 2017). In this study, treatment of the UC mice with MLF changed the abundance and diversity of gut microbiota, indicating that the MLF could alleviate symptoms of UC inflammation by changing the abundance and diversity of gut microbiota.

To comprehensively consider these results, we suggested that the alleviating effect of MLF on UC might be attributable to the combined effects of inflammation, intestinal epithelial barrier, and gut microbiota. The reduction of inflammation could restore the expression of tight junction proteins. The tight junction proteins could improve the integrity of the intestinal epithelial barrier and maintain normal gut microbiota, and vice versa. Compared with other *L. fermentum*, the synergy effect of the three physiological characteristics might be the reasons why MLF had a rapid alleviate of the UC phenotype (Geier et al., 2007; Santiago-Lopez et al., 2018; Zhou et al., 2019b).

Many studies had shown different mechanisms of anti-inflammatory effects in probiotics, but it was unclear what was the active ingredients in the metabolites of probiotics. The metabolites of probiotics contained a variety of components, including exopolysaccharides (Tallon et al., 2003; Santiago-Lopez et al., 2018), protein (Chon et al., 2010), lipids (Frick et al., 2007), and so on. Therefore, we carried out the anti-inflammatory response of these three substances in this article. It was worth noting that both exopolysaccharides and protein reduced the release of IL-6 and IL-1β, but not the lipids. It was reported that the exopolysaccharides produced by *Lactobacillus plantarum* YW11 could effectively alleviate the symptoms of IBD by recovering the microbial diversity and improving immunity of the host to reduce the risk of IBD symptoms (Min et al., 2020). In 2009, Chon et al. (2010) demonstrated that protein fractions derived from metabolites of *Lactobacillus plantarum* 10hk2 could regulate the anti-inflammatory response in RAW 246.7 cells stimulated by LPS. The root cause was that protein could inhibit the production of pro-inflammatory cytokines. In addition, the studies also reported that exopolysaccharides could induce more TNF-a production (Chon et al., 2010), Figure 9F also reflected this phenomenon. We speculated that the increase of TNF-α content may also be related to exopolysaccharides. In addition, although IL-6 was the downstream factor of TNF-α, signal transduction pathways in cells were network-like. A factor could be regulated by multiple pathways simultaneously. MLF was a metabolic product of bacteria, not a single substance. Thus, MLF could target multiple signaling pathways. We speculated that the increase of TNF-α was the results of multiple signaling pathway regulations. The further research was needed for the specific situation.

## Conclusion

Collectively, we screened a new *L. fermentum* F-B9-1 whose metabolites could ameliorate DSS-induced UC symptoms by decreasing the level of pro-inflammatory cytokines IL-6 and IL-1β, maintaining the expression of intestinal tight junction proteins and recovering the normal gut microbiome. Furthermore, the exopolysaccharides and protein from MLF might be the active constituents for the inflammation inhibition. Based on our findings, MLF might be a potential probiotic agent for ameliorating colitis.

## Data Availability Statement

The datasets presented in this study can be found in online repositories. The name of the repository and accession number can be found at: NCBI; PRJNA821927.

## Ethics Statement

The animal study was reviewed and approved by Animal Experiment Ethics Committee of Qilu University of Technology.

## Author Contributions

LS and LZ contributed to conception and design of the study. FM organized the database, performed the statistical analysis, and wrote the first draft of the manuscript. All authors contributed to the article and approved the submitted version.

## Funding

This work was supported by Shandong Taishan leading talent project (grant numbers LJNY202015 and tscy20180507), Spring Industry Leader Talent Support Plan (grant numbers 2017035 and 2019042), Key R&D Program of Shandong Province (grant number 2019QYTPY024); Yantai Development Zone Science and Technology Leading Talents Project (grant number 2020CXRC4); Science, Education and Industry Integration and Innovation Pilot project of Qilu University of Technology (Shandong Academy of Sciences; grant numbers 2020KJCYJ01 and 2020KJC-GH10), National key plan “science and Technology to help the economy” special project (grant number SQ2020YFF0401390), and University, Government, Industry, Research Collaborative Innovation Fund project (2020-CXY45).

## Conflict of Interest

LS was employed by the company Shengshengxiangrong (Shandong) Biotechnology Co., Ltd. KL and BL were employed by the company Jinan Hangchen Biotechnology Co., Ltd. LZ was employed by the company Shandong Chenzhang Biotechnology Co., Ltd.

The remaining authors declare that the research was conducted in the absence of any commercial or financial relationships that could be construed as a potential conflict of interest.

## Publisher’s Note

All claims expressed in this article are solely those of the authors and do not necessarily represent those of their affiliated organizations, or those of the publisher, the editors and the reviewers. Any product that may be evaluated in this article, or claim that may be made by its manufacturer, is not guaranteed or endorsed by the publisher.
